# The Influence of Socio-economic, Behavioural and Environmental Factors on *Taenia* spp. Transmission in Western Kenya: Evidence from a Cross-Sectional Survey in Humans and Pigs

**DOI:** 10.1371/journal.pntd.0004223

**Published:** 2015-12-07

**Authors:** Nicola A. Wardrop, Lian F. Thomas, Peter M. Atkinson, William A. de Glanville, Elizabeth A. J. Cook, C. Njeri Wamae, Sarah Gabriël, Pierre Dorny, Leslie J. S. Harrison, Eric M. Fèvre

**Affiliations:** 1 Geography and Environment, University of Southampton, Southampton, United Kingdom; 2 Centre for Immunity, Infection and Evolution, University of Edinburgh, Edinburgh, United Kingdom; 3 International Livestock Research Institute, Nairobi, Kenya; 4 Faculty of Science and Technology, Lancaster University, Lancaster, United Kingdom; 5 Faculty of Geosciences, University of Utrecht, Utrecht, The Netherlands; 6 School of Geography, Archaeology and Palaeoecology, Queen's University Belfast, Belfast, United Kingdom; 7 Centre for Microbiology Research, Kenya Medical Research Institute, Nairobi, Kenya; 8 Mount Kenya University, Thika, Kenya; 9 Unit of Veterinary Helminthology, Institute of Tropical Medicine, Antwerp, Belgium; 10 Royal (Dick) School of Veterinary Studies, University of Edinburgh, Edinburgh, United Kingdom; 11 Institute of Infection and Global Health, University of Liverpool, Liverpool, United Kingdom; Universidad Nacional Autónoma de México, MEXICO

## Abstract

*Taenia* spp. infections, particularly cysticercosis, cause considerable health impacts in endemic countries. Despite previous evidence of spatial clustering in cysticercosis and the role of environmental factors (e.g. temperature and humidity) in the survival of eggs, little research has explored these aspects of *Taenia* spp. epidemiology. In addition, there are significant gaps in our understanding of risk factors for infection in humans and pigs. This study aimed to assess the influence of socio-economic, behavioural and environmental variables on human and porcine cysticercosis. A cross-sectional survey for human taeniasis (*T*. *solium* and *T*. *saginata*), human cysticercosis (*T*. *solium*) and pig cysticercosis (*T*. *solium*) in 416 households in western Kenya was carried out. These data were linked to questionnaire responses and environmental datasets. Multi-level regression was used to examine the relationships between covariates and human and porcine cysticercosis. The HP10 Ag-ELISA sero-prevalence (suggestive of cysticercosis) was 6.6% for humans (95% CI 5.6%–7.7%), and 17.2% for pigs (95% CI 10.2%–26.4%). Human taeniasis prevalence, based on direct microscopic observation of *Taenia* spp. eggs (i.e. via microscopy results only) was 0.2% (95% CI 0.05%–0.5%). Presence of *Taenia* spp. antigen in both humans and pigs was significantly associated with a range of factors, including positive correlations with land cover. The presence of HP10 antigen in humans was correlated (non-linearly) with the proportion of land within a 1 km buffer that was flooding agricultural land and grassland (odds ratio [OR] = 1.09 and 0.998; p = 0.03 and 0.03 for the linear and quadratic terms respectively), gender (OR = 0.58 for males compared to females, p = 0.02), level of education (OR = 0.62 for primary level education versus no formal education, p = 0.09), use of well water for drinking (OR = 2.76 for those who use well water versus those who do not, p = 0.02) and precipitation (OR = 0.998, p = 0.02). Presence of *Taenia* spp. antigen in pigs was significantly correlated with gender and breeding status of the pig (OR = 10.35 for breeding sows compared to boars, p = 0.01), and the proportion of land within a 1 km buffer that was flooding agricultural land and grassland (OR = 1.04, p = 0.004). These results highlight the role of multiple socio-economic, behavioural and environmental factors in *Taenia* spp. transmission patterns. Environmental contamination with *Taenia* spp. eggs is a key issue, with landscape factors influencing presence of *Taenia* spp. antigens in both pigs and humans.

## Author Summary

*Taenia* spp. can cause tapeworm infections in the human gut, and infection with the larval stage of *Taenia solium* (cysticercosis) can lead to serious outcomes, such as epilepsy. Transmission occurs in areas with poor sanitation and a lack of adequate meat inspection practices, although there are still gaps in our understanding of how socio-economic, behavioural and environmental factors influence the occurrence of these parasites. Humans and pigs residing in 416 households in an area of western Kenya were tested for larval stage infections (cysticercosis). Statistical methods were applied to examine the relationships between a range of socio-economic, behavioural and environmental factors and disease occurrence. The results indicate that several factors, including land cover, influence the distribution of cysticercosis infections in humans and pigs. Further research in this area may provide significant understanding regarding the influence of environmental drivers on *Taenia* spp. infections, delivering evidence to support the targeting of disease control activities.

## Introduction

Taeniasis and cysticercosis are two human disease outcomes caused by parasites in the genus *Taenia*: taeniasis is infection with an adult tapeworm, while cysticercosis is infection with larval stages (of *Taenia solium*) in body tissues. Taeniasis, acquired via ingestion of undercooked meat containing the larval stage of the parasite, is not a significant health problem, generally producing asymptomatic infections or mild symptoms. However, carriers of *T*. *solium* tapeworms are a source of infection for human cysticercosis, which can produce long-term health problems. The transmission of *Taenia* spp. from a tapeworm carrier occurs via the shedding of eggs in faeces, followed by their ingestion by animal hosts (e.g. pigs for *T*. *solium* and cattle for *Taenia saginata*) and subsequent development into cysticerci [1]. Humans can also act as a ‘dead-end’ host for the larval stage of *T*. *solium*: accidental ingestion of tapeworm eggs results in the development of cysticerci in various tissues. The development of cysticerci in the central nervous system causes the most serious form of the disease, neurocysticercosis, which can produce neurological symptoms including seizures and is thought to be the leading cause of adult-onset epilepsy, responsible for up to one third of acquired epilepsy in *T*. *solium* endemic areas [2].

Due to the role of faecal contamination in transmission, *Taenia* spp. infections are common in developing countries with inadequate sanitation [3]. Recent pig population growth in some regions, including parts of sub-Saharan Africa, has led to concerns over increasing incidence of taeniasis, cysticercosis, and neurocysticercosis [3]. Despite the recognition of *T*. *solium* as a significant health problem, there are still few data available regarding its incidence and spatial distribution; substantial gaps in our epidemiological understanding; and a lack of reliable diagnostic tools for field use [1,4]. Thus, taeniasis and cysticercosis are considered to be neglected tropical diseases [4].

Increased risk of cysticercosis in pigs and humans has been associated to a lack of latrine availability or use [3,5,6], and free-ranging pig husbandry practices [7–9], highlighting the importance of environmental contamination. A single tapeworm can release up to 300,000 eggs per day, but the influences of environmental factors on egg survival have not been well studied [10]. Egg survival is influenced by temperature and humidity, with tropical regions being particularly suitable for transmission [10]. Surface moisture and humidity are thought to be the main constraining factors for *Taenia* spp. eggs in the environment: the eggs are vulnerable to desiccation and survival is greatly reduced under dry conditions, regardless of temperature [11]. Mechanical spatial spread of eggs can also occur via movement in streams, rivers or flood waters and via the activity of dung beetles.

Epidemiological analysis of several helminth species (e.g. hookworm, roundworm *Ascaris lumbricoides* and whipworm *Trichuris trichiura*) whose transmission cycles involve environmental contamination and subsequent egg maturation in the soil, has highlighted the role of environmental factors, including rainfall, temperature and vegetation cover, in the spatial distribution of these infections [12,13]. Soil-related factors (e.g. soil type) and land cover are also associated with helminth distributions, due to effects on soil humidity and egg maturation [14]. Although the lifecycle of *Taenia* spp. does not require egg maturation in the soil, egg survival and on-going transmission patterns are likely to exhibit correlations with environmental and climatic variables. Spatial analyses have demonstrated significant clustering of taeniasis, porcine cysticercosis and human cysticercosis, with evidence of aggregation of human and porcine cysticercosis cases within close proximity to human tapeworm carriers [15–18]. However, the extension of these analyses to encompass environmental covariates has not been carried out, despite the potential value.

This research aimed to assess the hypothesis that spatial clustering of cysticercosis is the result of a combination of (a) localised transmission cycles giving rise to spatial aggregations and (b) the impact of environmental conditions on egg survival and, thus, onward transmission. The influence of socio-economic, behavioural and environmental variables was assessed for human and porcine cysticercosis. Evidence for the influence of environmental factors on the distribution of *Taenia* spp. infections may provide the basis for further epidemiological research, to support the development and targeting of control programmes.

## Methods

### Ethics statement

Ethical approval was granted by the Kenya Medical Research Institute Ethical Review Board (SC1701; human sample collection), the Animal Welfare and Ethical Review Body (AWERB) at The Roslin Institute, University of Edinburgh (approval number AWA004; pig sample collection) and the University of Southampton ethics review committee (ID 1986; secondary data analysis). Written informed consent was obtained for all study participants and individual data was stored without identifiable information for the purposes of the analysis presented in this manuscript, to ensure anonymity.

### Cross-sectional survey

**Fig 1:**
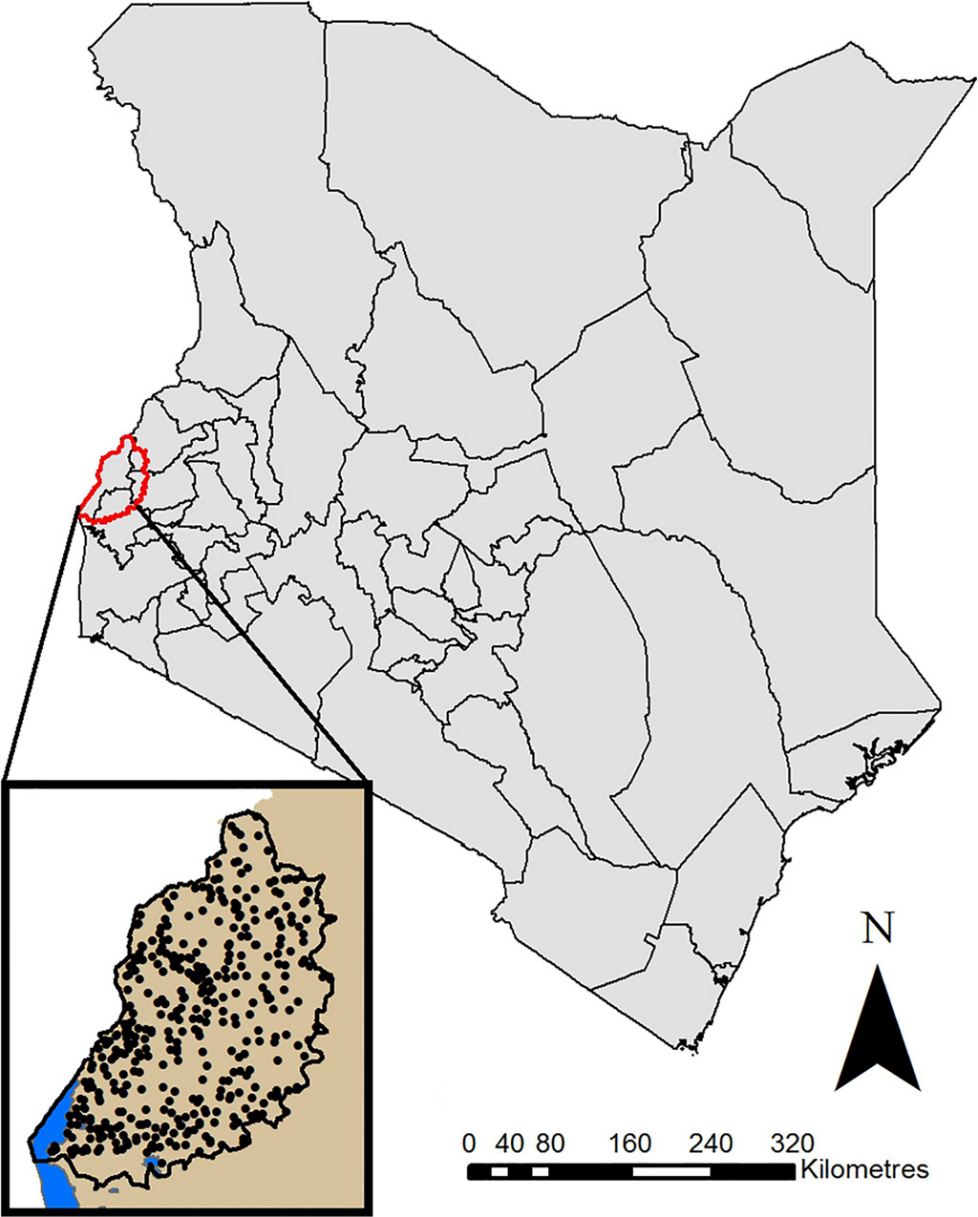
Map of the study area. Map of Kenya highlighting location of the study area (red outline), with inset map of study area illustrating the household locations.

The research focused on an area of western Kenya, as illustrated in Fig 1, which was selected as representative of areas at high risk of zoonotic diseases in the Lake Victoria crescent area of East Africa. The population density is approximately 500 per km^2^ and subsistence agriculture (mixed crop-livestock) is the predominant occupation, with the cattle population outnumbering the pig population. The study population includes several ethnicities, with the majority from the Luhya, Luo, Teso and Samia ethnic groups. The climate is bimodal, with rainy seasons from March to May and August to November, and an annual average temperature of approximately 22°C (range 14°C to 30°C) [19]. Data from a cross-sectional survey examining a range of zoonotic and non-zoonotic diseases (including *Taenia* spp.) in 416 households, carried out between July 2010 and July 2012, were used. Taeniasis detection was carried out for human participants using microscopy (sensitivity 28.6% to 52.5%; specificity 85.7% to 99.9% [20,21]) and copro-antigen ELISA (sensitivity and specificity of 98% and 99.1%, respectively [20]), which identify current *Taenia* spp. infections. It should be noted that these methods detect both *T*. *solium* and *T*. *saginata* infections, but cannot differentiate them. Detection of *Taenia* spp. HP10 antigen (which is suggestive of cysticercosis) was carried out for human participants and pigs utilising the HP10 antigen ELISA (sensitivity 44.4% to 84% for porcine sera and 75% to 84.8% for human sera; specificity 45% to 100% for porcine sera and 94% to 96.5% for human sera [22–25]). A detailed description of the survey protocol and diagnostic methods is provided in S1 File. Individual infection status data was linked with covariates at the individual (e.g. age) and household (e.g. presence of a latrine) levels, including questionnaire responses and geographically linked datasets, as listed in Table 1. Further information regarding the covariate datasets used is provided in S2 File.

**Table 1:** Covariates used in the analysis of *Taenia* spp. antigen presence in human and porcine sera: Covariates used for each analysis are marked with an X.

		Human	Porcine
**Individual human variables**	Age group	X	
	Gender	X	
	Ethnic background	X	
	Religion	X	
	Education	X	
	Eat beef	X	
	Eat beef frequency	X	
	Eat pork	X	
	Eat pork frequency	X	
	Latrine use	X	
**Individual pig variables**	Age group		X
	Gender		X
	Origin		X
	Breeding status		X
**Household level variables**	Latrine in compound	X	X
	Latrine type	X	X
	Evidence of latrine use	X	X
	Previous village flooding	X	X
	Water source	X	X
	% agricultural land and grassland*	X	X
	% flooding land*	X	X
	% flooding agricultural land and grassland*	X	X
	% swamp*	X	X
	% woodland and shrubs*	X	X
	% vegetated land*	X	X
	% water bodies*	X	X
	Soil sand content	X	X
	Water pH	X	X
	Mean temperature	X	X
	Precipitation	X	X
	Elevation	X	X
	Population density	X	X

*Land cover variables were calculated as the percentage of land within a 1 km buffer that consisted of each land cover class.

### Statistical analysis

Based on a suspected overestimation of human taeniasis (see results and discussion for further information), this outcome was not included in further statistical analyses. Due to the clustered nature of the data, the between-household variability in the odds of occurrence for each outcome was assessed. For each outcome, a single-level and a multilevel logistic regression model (including household level random effects) were fit to the data with no covariates. Likelihood ratio tests were used to assess the null hypothesis of no difference in the outcome between households. Where the null hypothesis was rejected (presence of *Taenia* spp. antigen in human sera), a multilevel model was used; where the null hypothesis was not rejected (presence of *Taenia* spp. antigen in porcine sera) a single-level model was applied.

For the land cover and precipitation covariates, the functional forms of associations with each of the outcome variables were assessed using univariable logistic regression analysis. Models including the covariates as linear, quadratic, square root and log terms were fitted to the data. Model comparison, based on AIC values and Chi-squared tests, was carried out to assess whether the non-linear terms improved the fit of the model. Where a non-linear term resulted in a statistically significant (*p*-value of 0.05 or less) improvement in model fit (reduction in AIC), this term was used rather than the linear term in further analysis.

Following the assessment of the functional form of associations, univariable logistic regression models were used to assess the significance of each of the covariates indicated in Table 1. This was followed by multivariable logistic regression including all the individual level covariates with a *p*-value of 0.1 or less in the univariable analysis. Next, household level covariates with a (univariable analysis) *p-*value of 0.1 or less were included, one at a time. At all steps, covariates no longer significantly associated with the outcome were removed. Where covariates were correlated with one another, covariate selection was performed based on understanding of the transmission cycle and comparison of AIC values.

A receiver operating characteristic (ROC) curve was created using observed outcomes and fitted values for each multivariable model, and the area under the ROC curve (AUC) calculated as a measure of model fit (AUC = 1 indicates perfect prediction; AUC = 0.5 indicates a prediction which performs no better than random). All statistical analyses were carried out in the R statistical software with *lme4* (multilevel models) and *stats* (single level models) packages. The corresponding author had full access to all the data in the study and had final responsibility for the decision to submit for publication. See S1 Checklist for the STROBE checklist for this cross-sectional study.

## Results

### Cross-sectional survey

416 households were recruited into the study with a minimum of 1 occupant and a maximum of 21, giving a mean household population of 5.1. Of the 416 households, 56.2% kept cattle and 16.9% kept pigs. Over half of pig keeping households (65%) kept only one pig and the mean herd size was 2.6. In total, 2113 humans (approximately 0.15% of the human population within the overall study area) and 93 pigs were included in the study, with stool samples obtained from 2057 humans (for taeniasis detection) and serum from 2092 humans and 93 pigs (for cysticercosis detection). Females accounted for 53.6% of the human study population.

**Fig 2:**
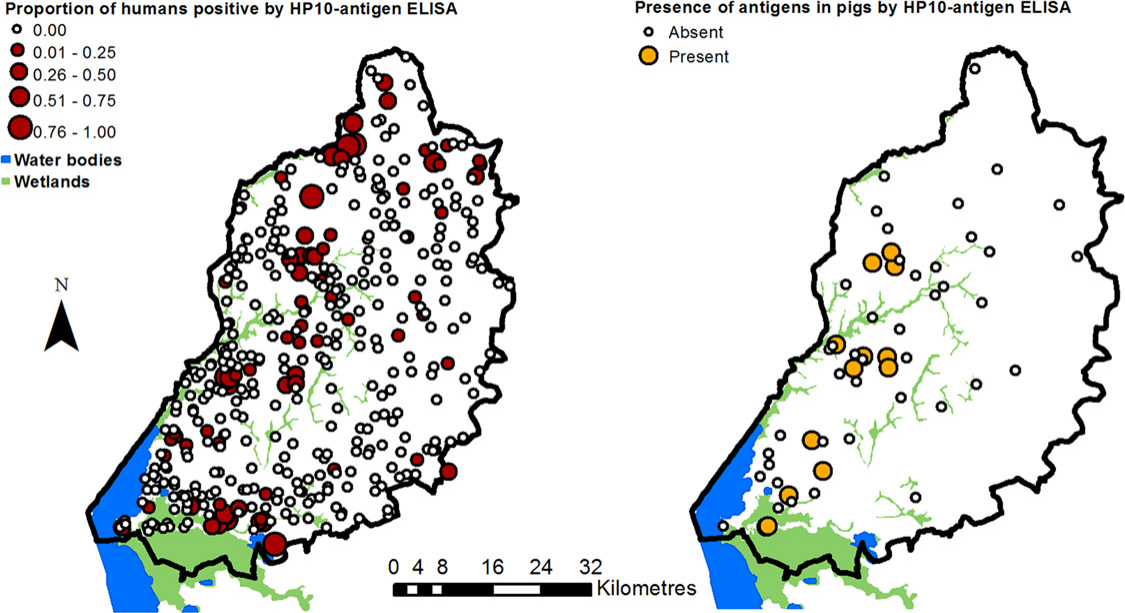
Household sampling results for HP10-antigen detection. The percentage of the household residents tested who were found to be positive by HP10-antigen ELISA is displayed for the human results, but due to the relatively small numbers of pigs tested, household level presence or absence of antigen in pigs is displayed rather than percentage positive.

The correlation between the two diagnostic methods for taeniasis (microscopy and copro-antigen ELISA) was zero: four participants were positive for taeniasis via microscopy only, 397 were positive for taeniasis via copro-antigen detection only and none were positive using both methods. The prevalence of taeniasis, based on direct observation of *Taenia* spp. eggs (i.e. using only the microscopy results), was 0.2% (95% confidence interval [CI]: 0.05%–0.5%, note that this includes both *T*. *saginata* and *T*. *solium* and the methods used cannot differentiate between them). Based on the lack of correlation between the two diagnostic methods, and unexpectedly high number of positive results, the taeniasis results were not used in further statistical analyses as a precautionary measure. The prevalence of *Taenia* spp. antigen (suggestive of cysticercosis) was 6.6% in humans (95% CI: 5.6%–7.7%) and 17.2% in pigs (95% CI: 10.2%–26.4%). *Taenia* spp. antigen was detected in at least one householder in 74 of the 416 households. Of the 55 pig-keeping households, 13 had at least one pig with detected *Taenia* spp. antigen. See Fig 2 for the observed spatial distribution of the disease outcomes and Tables 2 and 3 for descriptive data for prevalence of *Taenia* spp. antigen detection in human and porcine sera respectively.

**Table 2:** Descriptive data for prevalence of *Taenia* spp. antigen (suggestive of cysticercosis) in humans for categorical covariates at levels 1 and 2.

		Group	HP10 negative	HP10 positive	Prevalence
**Level 1 covariates**	**Age group**	**5–14**	815	59	6.8%
		**15–24**	362	21	5.5%
		**25–39**	348	23	6.2%
		**40–59**	288	18	5.9%
		**60 +**	141	17	10.8%
	**Gender**	**Female**	1036	86	7.7%
		**Male**	918	52	5.4%
	**Ethnic origin**	**Luhya**	999	56	5.3%
		**Luo**	427	36	7.8%
		**Samia**	236	19	7.5%
		**Teso**	277	26	8.6%
		**Other**	15	1	6.3%
	**Religion**	**Muslim**	39	2	4.9%
		**Christian**	1870	136	6.8%
		**None**	13	0	0%
		**Other**	31	0	0%
	**Education**	**None**	338	31	8.4%
		**Primary**	1364	90	6.2%
		**Secondary**	200	12	5.7%
		**Above**	48	4	7.7%
	**Eat beef**	**No**	269	18	6.3%
		**Yes**	1683	120	6.7%
	**Frequency of beef**	**Weekly**	645	51	7.3%
		**Less often**	1020	68	6.3%
		**Never**	289	19	6.2%
	**Eat pork**	**No**	671	47	6.5%
		**Yes**	1283	91	6.6%
	**Frequency of pork**	**Weekly**	281	29	9.4%
		**Less often**	1002	62	5.8%
		**Never**	671	47	6.5%
	**Frequency using latrine**	**Always**	1234	95	7.1%
		**Frequently**	365	17	4.5%
		**Sometimes**	239	16	6.3%
		**Never**	113	10	8.1%
**Level 2 covariates**	**Latrine in compound**	**No**	394	35	8.2%
		**Yes**	1560	103	6.2%
	**Latrine type**	**Completely closed**	413	37	8.2%
		**Partially closed**	1067	62	5.5%
		**Open pit**	80	4	4.8%
		**None**	394	35	8.2%
	**Evidence of latrine use**	**No**	38	4	9.5%
		**Yes**	1522	99	6.1%
		**No latrine**	394	35	8.2%
	**Recent village flooding**	**No**	1608	98	5.7%
		**Yes**	346	40	10.4%
	**Pig keeping**	**No**	1539	124	7.5%
		**Yes**	415	14	3.3%
	**Well water**	**No**	1686	101	5.7%
		**Yes**	268	37	12.1%
	**Use roof water**	**No**	1157	72	5.9%
		**Yes**	797	66	7.6%
	**Use river water**	**No**	1574	115	6.8%
		**Yes**	380	23	5.7%
	**Use piped water**	**No**	1810	134	6.9%
		**Yes**	144	4	2.7%
	**Use dam/pond water**	**No**	1870	136	6.8%
		**Yes**	84	2	2.3%
	**Use borehole water**	**No**	1045	78	6.9%
		**Yes**	909	60	6.2%
	**Use spring water**	**No**	1076	99	8.4%
		**Yes**	878	39	4.3%

**Table 3:** Descriptive data for prevalence of *Taenia* spp. antigen (suggestive of cysticercosis) in pigs for categorical covariates.

Covariates	Group	HP10 negative	HP10 positive	Prevalence
**Age group**	**<4 months**	8	1	0%
	**4–12 months**	61	11	15.3%
	**>12 months**	8	5	38.5%
**Gender**	**Female**	42	10	19.2%
	**Male**	35	6	14.6%
**Origin**	**Born in homestead**	20	5	20.0%
	**External**	57	11	16.2%
**Gender and breeding status**	**Male**	35	6	14.6%
	**Non-breeding sow**	39	6	13.3%
	**Breeding sow**	3	4	57.1%
**Sheep kept**	**No**	58	8	12.1%
	**Yes**	19	8	29.6%
**Goats kept**	**No**	36	7	16.3%
	**Yes**	41	9	18.0%
**Latrine in compound**	**No**	8	1	11.1%
	**Yes**	69	15	17.9%
**Latrine type**	**Completely closed**	16	1	5.9%
	**Partially closed**	52	12	20.0%
	**Open pit**	1	1	50.0%
	**None**	8	1	11.1%
**Latrine scavenging**	**No latrine**	8	1	11.1%
	**No**	59	13	18.1%
	**Yes**	10	2	16.7%
**Recent village flooding**	**No**	69	14	16.9%
	**Yes**	8	2	20.0%
**Sell piglets**	**No**	24	2	7.7%
	**Yes**	51	13	20.3%
**Raise pigs for meat**	**No**	17	5	22.7%
	**Yes**	58	10	14.7%

### Statistical analysis

Significant between-household variability was observed in the prevalence of *Taenia* spp. antigen (suggestive of cysticercosis) in humans, (likelihood ratio test p<0.005), but not in pigs (likelihood ratio test p>0.05). Therefore, a multilevel model was applied for human data, and single-level model for porcine data.

The majority of environmental covariates, when included in a univariable model as non-linear terms, did not significantly improve model fit in comparison to inclusion as linear terms (Tables A and B in S3 File). A non-linear relationship (quadratic) was indicated between the percentage of land that was flooding agricultural and grassland, and presence of *Taenia* spp. antigen in humans (p = 0.04). Thus, subsequent analysis of human data included the percentage of land that was flooding agricultural and grassland as a quadratic term.

Univariable regression results are presented in Tables C to E in S3 File. Sanitation related covariates (frequency an individual uses a latrine; presence of latrine within household; type of latrine; evidence of latrine use; or evidence of scavenging around the latrine by pigs), which have previously been shown to be associated with cysticercosis occurrence, were not significantly correlated with the presence of *Taenia* spp. antigen in pigs or humans.

The results from the multivariable human model for presence of *Taenia* spp. antigen in humans (Table 4) indicate a smaller odds of antigen presence in males compared to females (OR = 0.58, p = 0.02) and in those with primary education or higher compared to those with no education (OR for primary level = 0.62, p = 0.09). The use of wells as a water source was associated with a higher odds of antigen presence (OR = 2.76, p = 0.02). The percentage of land that was flooding agricultural or grassland had a quadratic relationship with presence of antigen in humans: lower odds of antigen presence were associated with very low or very high percentages of this land cover class, while the odds of antigen presence were highest in areas with intermediate percentages of the land cover class (OR = 1.09 and 0.998; p = 0.03 and 0.03 for the linear and squared terms respectively). Precipitation was negatively associated with the outcome (OR = 0.998, p = 0.02). The final model for presence of *Taenia* spp. antigen in pigs (Table 5) indicated that breeding sows had significantly higher odds of antigen presence compared to male pigs (OR = 10.35, p = 0.01), and flooding agricultural land and grassland demonstrated a positive association with the outcome (OR = 1.04, p = 0.004).

**Table 4:** Multivariable model for presence of *Taenia* spp. antigen in humans. AUC = 0.96.

	Covariate	Category	Odds ratio (95% CI)	p-value
	**Intercept**		0.42 (0.03–5.34)	0.51
**Individual level covariates**	**Gender (female is ref)**	Male	0.58 (0.37–0.91)	0.02
	**Education (none is ref)**	Primary	0.62 (0.36–1.07)	0.09
		Secondary	0.70 (0.30–1.66)	0.42
		Above	0.74 (0.18–2.99)	0.67
**Household level covariates**	**Use well water (no is ref)**	Yes	2.76 (1.17–6.51)	0.02
	% flooding agricultural land and grassland*	NA	1.09 (1.01–1.17)	0.03
	**% flooding agricultural land and grassland** ^**2**^ *	NA	0.998 (0.996–0.999)	0.03
	**Precipitation (mm)**	NA	0.998 (0.996–0.999)	0.02

*Percentage of land within a 1 km buffer around the homestead which contains flooding agricultural land and grassland (included as a quadratic expression).

**Table 5:** Multivariable model for presence of *Taenia* spp. antigen in pigs. AUC = 0.77.

Covariate	Category	Odds ratio (95% CI)	p-value
**Intercept**		0.09 (0.03–0.24)	<0.005
**Gender/breeding status (male is ref)**	Non breeding sow	0.70 (0.17–2.58)	0.57
	Breeding sow	10.35 (1.72–70.84)	0.01
% flooding agricultural land and grassland*	NA	1.04 (1.01–1.07)	0.004

*Percentage of land within a 1 km buffer around the homestead which contains flooding agricultural land and grassland.

The AUC value was 0.96 for the human model, indicating excellent model fit, and 0.77 for the porcine model, indicating fair model fit.

## Discussion

Previous evidence has indicated spatial clustering of taeniasis and cysticercosis. This may be the result of localised transmission cycles; the impact of environmental conditions on egg survival; or a combination of these [15–18]. The results presented here indicate endemic transmission of *Taenia* spp. in the study area, and demonstrate significant associations between assumed cysticercosis in both pigs and humans (based on presence of *Taenia* spp. HP10 antigen in sera), and land cover, after accounting for other known risk factors. This supports the hypothesis that spatial heterogeneity in the distribution of infections may be influenced by environmental conditions, highlighting the interplay between socio-economic, behavioural and environmental factors in *Taenia* spp. infection risk in humans and pigs.

A recent review of previously published studies across Africa demonstrated taeniasis prevalence ranging from 0% to 8.7% (although these studies do not use a standardised diagnostic protocol) [1]. A higher prevalence of 13.15% has been reported from Ghana, based on detection by microscopy [26]. The detected prevalence of taeniasis based on direct observation of *Taenia* spp. eggs in this study was 0.02%, which lies within the previously reported range. However, the results from the copro-Ag ELISA suggested a far larger number of tapeworm carriers within the study population. The methods used for taeniasis detection do not allow differentiation of *Taenia* species, and so this prevalence estimate includes both *T*. *saginata* and *T*. *solium*. Previous research has highlighted that hyper-endemic transmission of *T*. *solium* (characterised by human cysticercosis prevalence of up to 27% and porcine prevalence up to 75%) can be associated with a taeniasis prevalence of less than 7%, and, in general, cysticercosis prevalence is higher than prevalence of taeniasis [27–29]. Based on our detected prevalence of *Taenia* spp. antigen (suggestive of cysticercosis) of 6.6% in humans and 17.2% in pigs, along with a lack of correlation between microscopy and copro-Ag ELISA results in this study, we suspect that the copro-antigen ELISA results were inaccurate, and thus, these data were not included in further analyses. More accurate assessment of taeniasis prevalence in this setting is warranted to ensure an accurate picture of the epidemiology of *Taenia* spp. is available. This could be achieved by providing antihelminitic treatment to those with a positive copro-antigen test, followed by assessment of stools for expelled tapeworms. However, this was not feasible to achieve within the described study given the broad range of infectious diseases targeted (i.e. the study was not focussed solely on detection of *Taenia* spp.) and the fact that copro-Ag ELISA was carried out after the completion of the field work in our Nairobi laboratory on anonymised samples.

Detected prevalence of cysticercosis (or *Taenia* spp. antigen) varied from 0% to 21.6% in humans and from 0% to 56.7% in pigs from previous research across Africa (and in general, porcine prevalence was higher than human prevalence in individual countries), although again, these did not use standardised diagnostic protocols [1]. Prevalence of *Taenia* spp. antigens in this study population was 6.6% for humans and 17.2% for pigs, which fall within the ranges previously reported. In a nearby study site (also in western Kenya), a substantially higher prevalence of porcine cysticercosis (32.8%) was detected: no data on human prevalence was available from this study site [5]. The results from this study, in combination with previously published data, highlight substantial variation in the prevalence of cysticercosis across different regions. However, a scarcity of data and a lack of understanding of the spatial heterogeneity of *Taenia* spp. transmission remain.

Odds of *Taenia* spp. antigen presence in humans were smaller in those with any level of formal education when compared with those with no education. Lack of education is commonly associated with higher prevalence of infectious diseases, particularly those related to sanitation [30]. Females had larger odds of antigen presence compared to males, which may be related to the daily activities of females within the study population: women provide up to 75% of agricultural labour in smallholder farming in Kenya, which may result in increased exposure to faecal contamination in the environment [31]. A similar gender difference has been identified elsewhere [32], although this finding cannot be generalised to all settings [33].

The use of well water was positively correlated with antigen presence in humans, indicating that contamination of well water with faecal pollutants is common. Previous research in Tanzania has also demonstrated larger odds of cysticercosis in those consuming “unsafe” water [34]. Protected (or improved) water sources, such as well constructed boreholes, can prevent faecal contamination: the sides of the borehole can be lined and the top covered to prevent direct entry of surface water and other contaminants. Wells, although they may be improved (e.g. covered to prevent surface water influx), are generally at higher risk of contamination as they are often left uncovered or inadequately covered, allowing potentially contaminated surface water to enter. Wells are also shallow in comparison to boreholes, meaning that even when covered, surface water has a shorter duration of soil filtration before entering the well, increasing further the risk of contamination [35,36]. Within the study area, well water is more common in areas with frequent flooding, presumably due to the requirement of a high water table. This combination of flooding and vulnerable water supplies may enhance contamination, thus increasing the risk of infection. Flooding agricultural land and grassland was also (non-linearly) associated with presence of antigens in humans, indicating a role for landscape factors in cysticercosis. The eggs of *Taenia* spp. are highly susceptible to desiccation, suggesting this association may be related to varying soil humidity in different landscapes (e.g. soil humidity will be highest under vegetation and in areas that flood periodically) [11]. In addition, human activities vary in different types of landscape, thus, altering contamination and exposure: agricultural land and grassland are accessed more frequently by humans than, for example, woodland, enhancing the possibility of environmental contamination (those working in the field do not always use a latrine) and subsequent exposure to eggs. Flooding may also be related to the movement of eggs, with flood waters potentially resulting in contamination of land with eggs from elsewhere. Precipitation was also significantly, and negatively, associated with presence of *Taenia* spp. antigen in humans. Based on the previous discussion regarding flooding and access to ground water, this relationship is not as expected. The southern part of the study area (which is at the lowest elevation) experiences the least rainfall, but includes the largest proportion of flooding land and has a larger proportion of the population using groundwater sources, particularly well water. A possible explanation for the observed relationship is the action of overland water flow following precipitation leading to eggs being washed away, whereas flooding events may be associated with egg deposition.

In terms of presence of *Taenia* spp. antigen in pigs, breeding female pigs had significantly higher odds of antigen presence compared to male or non-breeding females. This may be due to a longer period of exposure in the household (breeding females will be retained for a longer period than pigs raised for sale or slaughter), although age group alone was not significantly associated with porcine cysticercosis. In addition, flooding agricultural land and grassland was positively associated with the outcome, indicating that this land cover class may act to promote survival of eggs in the environment or enhance the exposure of pigs to faecal material. As discussed previously, this land cover class represents areas which are likely to have high soil moisture contents, may experience contamination via the movement of pathogens during periods of flooding and are likely to have high levels of human activity, thus increasing the possibility of faecal contamination.

It is important to recognise that a positive HP10-antigen ELISA result is suggestive of the presence of a viable cyst in the host (i.e. cysticercosis), which may not have been recently acquired. Due to the short lifespan of pigs, a positive HP10-antigen ELISA result will relate to a relatively recent infection. However, a positive result in humans may relate to a historical infection since cysts can remain in a host for several years. In addition, a positive result does not necessarily indicate neurocysticercosis: this may relate to muscular, neuro- or ocular-cysticercosis. The results should also be interpreted with consideration of the performance of the diagnostic methods used. The sensitivity of HP10 antigen-ELISA has been estimated at between 44.4% and 84% for porcine sera and between 75% and 84.8% for human sera. The specificity has been reported as between 45% and 100% for porcine sera and between 94% and 96.5% for human sera [22–25]. This assay was found to have low cross-reactivity with other helminth infections, except for cross-reactivity with other *Taenia* species, including *Taenia hydatigena* [37]. There is a lack of empirical data regarding the prevalence of *T*. *hydatigena* in pigs in East Africa: its presence has been documented, but its prevalence is thought to be low, with a recent study in Tanzania indicating a prevalence of 6.6% in pigs [38–40]. Further validation of the HP10 antigen-ELISA for detection of porcine cysticercosis using pig necropsy as the gold-standard, within the study area, is currently being planned. It was not feasible to conduct this within the described study.

The results of statistical analysis relating to the presence of antigen in pigs are less certain than those relating to the presence of antigen in humans, based on the smaller sample size (93 pigs), which limits our ability to draw firm conclusions. Movements of pigs and humans have not been considered in this analysis, although these movements are of clear importance for exposure to infection: pigs within this study area have been found to scavenge for food within a mean home range area of 10,343 m^2^ [41]. The inclusion of land cover within 1 km of the household should partially deal with this limitation. The proximity to tapeworm carriers has also been identified as an important factor determining the occurrence of cysticercosis [15,16]. As this study was based on a sample of individuals from the study area, information on the locations of all tapeworm carriers was not available and, thus, it was not possible to include this aspect in our analysis.

Overall, these results provide a useful insight into the epidemiology of *Taenia* spp. infections in a rural community and highlight key areas where interventions should be targeted. The World Health Organization lists the interventions for control of taeniasis and cysticercosis as: preventive chemotherapy; diagnosis and treatment of taeniasis cases; improved health education; improved sanitation; improved pig husbandry; treatment of pigs; vaccination of pigs; and improved meat inspection and processing [42]. This research, in combination with an understanding of the transmission cycle of *Taenia* spp., indicates that environmental contamination by eggs is a key issue, with environmental factors influencing the potential for cysticercosis in pigs and humans; large-scale interventions to address the control of cysticercosis should thus consider ways of reducing contamination in the environment as a means of reducing transmission.

This research provides an initial view of the complex interplay between individual level factors, household level factors and environmental factors in the spatial distribution of *Taenia* spp. infections in humans and pigs, indicating roles for both (a) localised transmission and (b) the influence of environmental factors. In reaching these conclusions we acknowledge the limitations of the assay procedures employed: further investigations, particularly the direct verification of parasitic infection and species identification by a combination of antihelmintic treatment, tapeworm identification, PCR and the inspection of pig carcasses are warranted.

Further research describing the environmental determinants of tapeworm and cysticercosis over larger areas and in different ecological systems would deliver the potential to provide national or regional level spatial predictions of infection risk. Such outputs would significantly improve our understanding of geographical heterogeneity in taeniasis and cysticercosis and allow the implementation of geographically targeted interventions.

## Supporting Information

S1 Checklist STROBE checklist. (DOC)

S1 File Detailed description of survey protocol and diagnostic methods. (DOCX)

S2 File Detailed description of covariates used in the analysis. (DOCX)

S3 File Exploratory analysis results. (DOCX)
