# Correction: The Influence of Socio-economic, Behavioural and Environmental Factors on *Taenia* spp. Transmission in Western Kenya: Evidence from a Cross-Sectional Survey in Humans and Pigs

**DOI:** 10.1371/journal.pntd.0004394

**Published:** 2016-01-13

**Authors:** Nicola A. Wardrop, Lian F. Thomas, Peter M. Atkinson, William A. de Glanville, Elizabeth A. J. Cook, C. Njeri Wamae, Sarah Gabriël, Pierre Dorny, Leslie J. S. Harrison, Eric M. Fèvre

There are errors in the Author Contributions. The correct contributions are: Conceived and designed the experiments: LFT EMF. Performed the experiments: LFT WAdG EAJC. Analyzed the data: NAW. Contributed reagents/materials/analysis tools: CNW SG PD LJSH EMF. Wrote the paper: NAW LFT PMA WAdG EAJC CNW SG PD LJSH EMF
