# Supplementary material for: The Influence of Socio-economic, Behavioural and Environmental Factors on Taenia spp. Transmission in Western Kenya: Evidence from a Cross-Sectional Survey in Humans and Pigs
Source: PLoS Negl Trop Dis. 2015 Dec 7;9(12):e0004223. doi: 10.1371/journal.pntd.0004223 (PMC4671581; doi:10.1371/journal.pntd.0004223)
Supplement: S1 File — (DOCX) [file pntd.0004223.s002.docx]

**Supporting information 1:** Detailed description of survey protocol and diagnostic methods.

1. **Survey protocol**

A clustered sampling design was used, with 416 households selected randomly from the study region: the number of households selected per sub-location (the smallest administrative area in Kenya) was weighted by the cattle population of each sub-location (i.e. more households selected in sub-locations with more livestock), resulting in between one and ten households per sub-location. Spatial points were generated randomly within each sub-location, and the closest household was selected for inclusion in the study. The precise geographical coordinates for the study households were recorded using a handheld global positioning system. Following a procedure of informed consent, all humans older than 5 years (excluding women in the third trimester of pregnancy) and all pigs (excluding pregnant animals in their third trimester and suckling piglets) within the household were included as study participants and stool (for humans) and blood (for humans and pigs) samples were obtained. Questionnaires were also administered for all households relating to household level factors (e.g. water sources, livestock keeping; answered by a single individual per household), individual human level factors (e.g. demographic information) and individual pig level factors (e.g. age, sex).

Parasitological diagnosis of taeniasis was carried out via microscopy using the Kato Katz and Formal Ether concentration techniques for the visualisation of *Taenia* spp. eggs or proglottids in faecal samples, which would indicate the presence of an adult worm in the gut ([1](#_ENREF_1)). Copro-antigen ELISA was also carried out on faecal samples to allow the detection of *Taenia* spp. antigens, providing a supplementary method for taeniasis detection ([2](#_ENREF_2)). Individuals were recorded as *Taenia* spp. tapeworm carriers where a positive result was obtained from at least one method (Kato Katz, Formal Ether or copro-antigen ELISA). Human and pig blood samples were tested using an HP10 ELISA, which detects circulating *Taenia* spp. antigens ([3-5](#_ENREF_3)): individuals (humans and pigs) were recorded as having *Taenia* spp. antigens in sera (which is suggestive of cysticercosis) where the HP10 ELISA gave a positive result. For the purposes of analyses, the survey data were recorded as binary outcomes (positive or negative) for each outcome.

1. **Ethical approval**

Ethical approval was granted by the Kenya Medical Research Institute Ethical Review Board (SC1701; human sample collection), the Animal Welfare and Ethical Review Body (AWERB) at The Roslin Institute, University of Edinburgh (approval number AWA004; pig sample collection) and the University of Southampton ethics review committee (ID 1986; secondary data analysis). Informed consent was obtained for all study participants and individuals names were not recorded to ensure anonymity.

1. **HP10 Ag-ELISA for detection of cysticercosis infections**

This procedure was carried out at the International Livestock Research Institute (ILRI), Nairobi. The HP10 antigen was detected by Ag-ELISA as described previously by Harrison *et al* ([6](#_ENREF_6)). The procedure was as follows: 100µl of a 10µg/ml solution of 50% saturated (NH_4_) _2_S0_4_ precipitate McAb HP10 diluted in carbonate-bicarbonate buffer 9.6 pH (Sigma C3041) was added to each of the wells of a flat bottomed Immunlon® 4HBX ELISA plate and the plate was incubated overnight at 4°C. The wells were washed out twice with washing solution (0.9% NaCl with 0.05% Tween®20 (Sigma P1379)), 200µl of phosphate buffered saline (PBS) 7.3 pH (Sigma P4417) / 1% Bovine Serum Albumin (BSA) (Sigma A4503) / 0.05% Tween®20 was added to each of the wells and incubated at room temperature for 1 hour to block any non-reacted sites on the plate. The plates were then washed three times with washing solution. 100µl of undiluted sera was then added to each well, with each sample running in duplicate. The plate was incubated for 1 hour at 37°C, followed by emptying and washing three times. Biotinylated-McAb diluted 1:2,500 in PBS/BSA/Tween®20 was added at 100µl/well, covered and incubated for 1 hour at 37°C followed again by washing three times. Streptavidin Peroxiase (sigma S5512) conjugate diluted 1:10,000 (ie 0.1µg/ml) in PBS/BSA/Tween®20 added at 100µl per well, covered and incubated for 1 hour at 37°C. After a further three washes 100µl 3,3’, 5,5’- Tetramethylbenzidine substrate (Sigma T8665) substrate was then added to each well and incubated at room temperature for 15 minutes. The reaction was then stopped with 100µl of 0.2M H_2_SO_4_ per well and read at 450nm on an ELISA plate reader.

Five negative controls were run on each plate and these were obtained from an indoor farrow- finish unit in the UK, a country that is free from *T. solium* cysticercosis. For screening of human samples negative controls were obtained from the PI of this project. Two positive controls were also run for each plate and these were obtained from experimental infections of cattle with *T. saginata* provided by Dr Leslie Harrison.

A correction factor as provided by Dr Leslie Harrison (per. comms. LJSH) was used to correct for plate-to-plate and day-to-day variations, this correction factor refers to the first plate of a screening run performed with the same negative and positive control sera. Correction factor = P_0_-N_0_/Pt-Nt where

P_0_ =mean of positive control sera plate 1

N_0_  = mean of negative control sera plate 1

Pt = mean of positive control sera test plate

Nt = mean of negative control sera test plate

Applying the correction factor to every samples optical density (OD) provides a standardisation of samples across the full run.

Cut-off values were determined by using the mean corrected OD of all negative controls run during the full screen plus three standard deviations. Any sera sample with a corrected OD value over this cut off was counted as being positive.

1. **Copro Ag-ELISA for detection of taeniasis infections**

Samples were prepared by mixing equal volumes of faecal sample and Phosphate Buffered Saline (PBS), soaking for one hour with intermittent shaking. The samples were then centrifuged for 30 minutes at 2000g, and the faecal supernatent aliquoted for analysis.

The negative control panel consisted of 63 non-meat eating people from within the study site. All were negative for *Taenia* spp. eggs on microscopy, 26 of the negative controls had no GI parasites, of the remaining 37 samples all were positive for at least one GI parasite, including, *Schistosoma mansoni*, *Strongyloides* spp.*, Giardia* spp., *Ascaris* spp., *Entaemeba* spp., *Iodamoeba* spp. and hookworm. The positive control samples were collected from two self-reported *Taenia* spp. carriers who expelled an adult worm on treatment with Nicosamide (2g orally as a single dose), followed 2 hr later by a purgative dose of castor oil (4tsp). Negative and positve control samples were prepared as above. Each sample was run in duplicate, with 2 negative controls, 2 positive controls and duplicate substrate and conujate controls per plate to check for the validity of each plate run.

A flat bottomed Nunc Maxisorp® plate was coated for 1 hour at 37˚C with 100µl IgG polyclonal at 2.5µg/ml in 0.05M Carbonate-Bicarbonate coating buffer (Sigma-Aldrich, C3041), with 100µl coating buffer only in the substrate control. The plate was then washed once in PBS-0.05% Tween20® before blocking for 1hour at 37˚C with PBS-0.05% Tween20® with 2% heat inactivated New Born Calf Serum (Invitrogen, 16010-167). After blocking, the solution was emptied and the plate tapped to dry, following which 100µl of sample was added to each well with blocking buffer used in the substrate and conujate control wells.

The samples were incubated for 1hr at 37˚C before washing 5x in PBS-0.05 %Tween20®. Detection used 100µl of biotinilated polycolonal at 2.5µg/ml in blocking buffer with blocking buffer only in the substrate control wells. After 1hr incubation at 37˚C the plate was washed 5x in PBS-0.05%Tween20®. 100µl of streptavidin horseradish peroxidase (Jackson immunoresearch) at 1:10,000 dilution was added to each well and incubated for 1hr at 37˚C before a further 5x washes with PBS-0.05%Tween20®. 100µl of OPD (DAKO) was then added to each well and incubated for 15minutes at 30˚C in the dark before the reaction was stopped with 50µl 0.5M H_2_SO_4_. The plate was then read with an ELISA plate reader (BioTek Synergy-HT) at 492 and 655mm.

The mean optical density (OD) of a panel of negative controls (63) plus 3x standard deviation were used to calculate the cut off value of OD = 0.874. Samples whose mean OD value was over the cut-off were considered to be positive.

1. World Health Organization. Bench Aids for the Diagnosis of Intestinal Parasites. Geneva: World Health Organization, 1994.

2. Allan JC, Velasquez-Tohom M, Torres-Alvarez R, Yurrita P, Garcia-Noval J. Field trial of the coproantigen-based diagnosis of Taenia solium taeniasis by enzyme-linked immunosorbent assay. Am J Trop Med Hyg. 1996;54(4):352-6.

3. Harrison LJS, Garate T, Bryce DM, Gonzalez LM, Foster-Cuevas M, Wamae LW, et al. Ag-ELISA and PCR for monitoring the vaccination of cattle against Taenia saginata cysticercosis using an oncospheral adhesion protein (HP6) with surface and secreted localization. Trop Anim Health Pro. 2005;37(2):103-20.

4. Harrison LJ, Joshua GW, Wright SH, Parkhouse RM. Specific detection of circulating surface/secreted glycoproteins of viable cysticerci in Taenia saginata cysticercosis. Parasite Immunol. 1989;11(4):351-70.

5. Krecek RC, Michael LM, Schantz PM, Ntanjana L, Smith MF, Dorny P, et al. Prevalence of Taenia solium cysticercosis in swine from a community-based study in 21 villages of the Eastern Cape Province, South Africa (vol 154, pg 38, 2008). Vet Parasitol. 2011;183(1-2):198-200.

6. Harrison LJP, R.M. Taenia saginata and Taenia solium: reciprocal models. Acta Leidensia. 1989;57(2):143-52.
