# Supplementary material for: The Influence of Socio-economic, Behavioural and Environmental Factors on Taenia spp. Transmission in Western Kenya: Evidence from a Cross-Sectional Survey in Humans and Pigs
Source: PLoS Negl Trop Dis. 2015 Dec 7;9(12):e0004223. doi: 10.1371/journal.pntd.0004223 (PMC4671581; doi:10.1371/journal.pntd.0004223)
Supplement: S2 File — (DOCX) [file pntd.0004223.s003.docx]

**Supporting information 2:** Detailed description of covariates used in the analysis.

1. **Land cover classification**

A 10 km by 10 km grid was created in ArcMap to cover the study area. 14 grid cells were selected from this grid at random: the sublocation(s) which formed the major part of these 14 grid cells were used as sampling sites for training data collection, resulting in 24 sublocations for sample site collections. This was carried out to ensure a representative sample of locations was used in the training data collection.

Training data was collected during October 2012. A research assistant with thorough knowledge of the roads and land cover within the study area was employed to assist with the land cover training data collection. During training data collection, the research assistant guided us along roads and paths throughout the sublocations of interest. A GPS, linked to a laptop running ArcMap, allowed real time location tracking. Training data polygons were created in ArcMap, relating to the specified land cover classes (see below) that were passed on the route. Manual checking of training data polygons was carried out via cross referencing GoogleEarth (where high resolution imagery was available) to minimise the impact of any training data collection errors. See Table S1 for a summary of the number of training areas recorded for each land cover class.

A separate set of training data were created using ArcMap: these identified areas of flooding and non-flooding land via visual inspection of ASTER imagery, from which areas of flooding land can be easily identified. This additional training data was selected due to the inherent difficulty in physically reaching flooded areas of land (e.g. swamps), to ensure good representation of flooding land for classification purposes. See Table S2 for a summary of the number of training areas recorded for flooding and non-flooding land. A random 10% sample of the training areas was selected for use in validation.

**Table A: Number of training areas for the main land cover classes, created during field work**

| **Class** | **Number of training areas** |
| --- | --- |
| Artificial and bare ground | 59 |
| Crops and grassland | 261 |
| Rice | 4 |
| Woodland and shrubland | 204 |
| Swamp | 40 |

**Table B: Number of training areas for flooding versus non-flooding land, created using visual assessment of ASTER imagery.**

| **Class** | **Number of training areas** |
| --- | --- |
| Flooding land | 23 |
| Non-flooding land | 18 |

- 1. **Remote sensing image preparation**

Two ASTER images from 11^th^ March 2010, which had no cloud cover, were selected for use in the classification. These were atmospherically corrected using the FLAASH module of ENVI, and then mosaicked to give a single image. See below for information on the scenes used and the settings used during atmospheric correction.

**Scenes information:**

ASTL1B 1003110806321003140170

Scene centre: 0.230217, 34.242193

Flight date: 11^th^ March 2010

Flight time: 08:06:32 08:06:23

ASTL1B 1003110806231003140169

Scene centre: 0.765325, 34.355913

Flight date: 11^th^ March 2010

Flight time: 08:06:23

Radiance scale factor: 10

Sensor altitude: 705.00

Pixel size: 15 m

Average scene altitude: 1,300 m

Atmospheric model: Tropical, rural

Two Landsat images were also selected for use: one from 3^rd^ July 2011 (Landsat 5 TM sensor) and one from 15^th^ December 2010 (Landsat 7 ETM+ sensor). The Landsat ETM+ image was affected by the scan line corrector failure, resulting in a striped image, therefore, this image was gap filled using a third Landsat image (fill image) from 29^th^ November 2010. Each of these three Landsat images were atmospherically corrected using the FLAASH module of ENVI. See below for information on the scenes used, the bands available and the settings used during atmospheric correction. Following atmospheric correction, the 15^th^ December Landsat image was gap-filled using local histogram matching with the fill image in ENVI (using the Landsat gap-filling algorithm). For clarity, the July Landsat image will be referred to as Jul Landsat and the December image as Dec Landsat.

Landsat 5 TM 06020110703

Scene centre: -0.00949955, 34.3042565

Flight date: 3^rd^ July 2011

Flight time: 07:44:21

Sun elevation: 52.375

Landsat ETM+ 06020101215 (SLC off)

Scene centre: 0.00542835, 34.29886655

Flight date: 15^th^ December 2010

Flight time: 07:48:10

Sun elevation: 54.646

Fill image Landsat ETM+ 06020101129

Scene centre: 0.00407185, 34.3002157

Flight date: 29^th^ November 2010

Flight time: 07:48:02

Sun elevation: 56.954

Radiance scale factor: 10

Sensor altitude: 705.00

Pixel size: 30 m

Average scene altitude: 1,300 m

Atmospheric model: Tropical, rural

The images selected cover March 2010 (start of the long rains), July 2011 (end of the long rains) and December 2010 (start of the dry season). These were selected to allow optimal differentiation of different land cover classes by providing coverage during different seasons and different sections of the agricultural calendar.

- 1. **Classification procedure**

The land cover classification was carried out using eCognition Developer 8, which uses an object orientated classification approach (as opposed to a pixel-based classification). Segmentation was carried out using the ASTER image only (as this image had the highest spatial resolution – 15 m): the image was segmented in a hierarchical manner using scales of 50 and 15 to produce two levels with varying object sizes (scale 50 produces larger objects than scale 15), called level 1 (scale 50) and level 2 (scale 15). The homogeneity criteria were set as 0.8 for colour and 0.2 for shape, with the compactness parameter set at 0.5.

The classification used a hierarchical approach, as demonstrated in Figure S1 below. Four main classification steps were carried out as part of this hierarchical approach:

1. Classify water bodies and land areas (level 1)
2. Classify vegetated and non-vegetated land
3. Classify flooding and non-flooding land
4. Classify specific land cover classes (level 2)


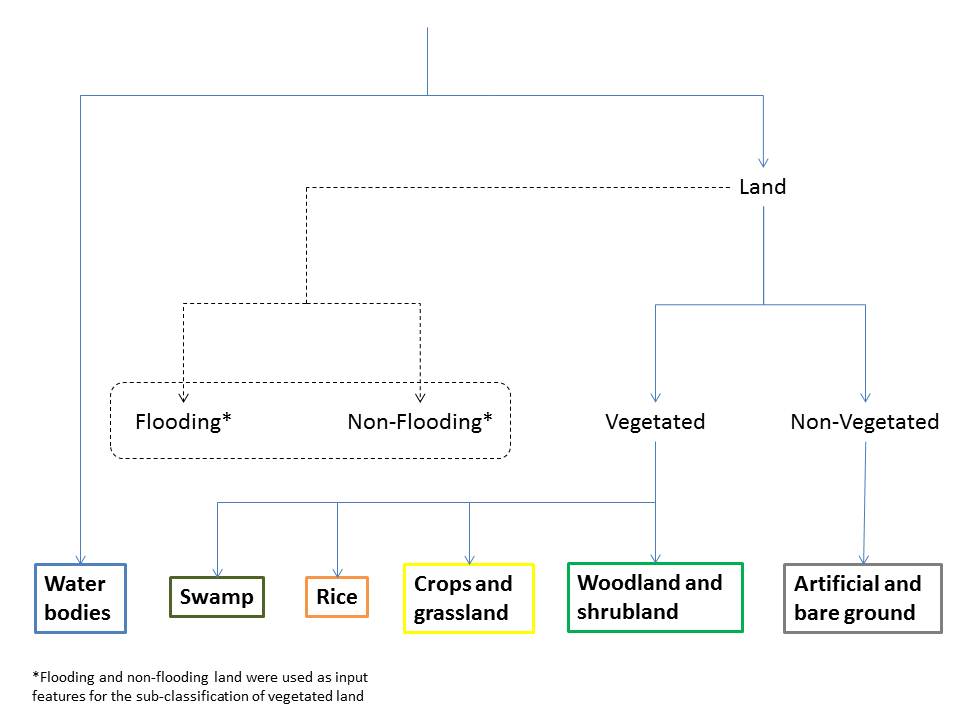


**Figure A: Diagram of the hierarchical classification procedure used.**

- - 1. **Water bodies and land areas – classification at level 1**

No training areas were used in the classification of water bodies versus land areas: visual interpretation was used to identify the most suitable threshold values to classify water bodies (rivers and lakes). Lake was classified as objects with mean ASTER band 3 <1020. River was classified as objects with mean Jul Landsat band 5 < 1500 and contrast to neighbouring pixels ASTER band 3 < -350. These classes were grouped together into a single “water bodies” class. A copy of level 1 with the water body classification was created to allow subsequent classification of the land areas into flooding and non-flooding land, and vegetated and non-vegetated land. To clarify – it is possible for flooding land to be either vegetated or non-vegetated and vice versa.

- - 1. **Flooding and non-flooding land – classification at level 1**

The flooding land training areas were imported into eCognition and used to create samples (overlap parameter set to 0.5). Nearest neighbour classification was performed on the objects which were not classified as water bodies in step 1 using the features: mean and absolute mean difference to neighbours (elevation); mean (ASTER band 2); mean of inner border (Jul Landsat band 5); and mean of outer border (ASTER bands 2 and 3). A subsequent filtering step was carried out to remove noise from the classification: objects classified to flooding land were merged together and if the resulting object was less than 600 pixels they were reclassified as non-flooding.

- - 1. **Vegetated versus non-vegetated areas – classification at level 1**

This classification used the copy of the water bodies classification layer (e.g. this was carried out independently of the flooding and non-flooding land classification). The training areas recorded during fieldwork were reclassified as either vegetated or non-vegetated and imported into eCognition. These were used to create samples for the classification (overlap parameter set to 0.1). Nearest neighbour classification was performed on the objects which were not classified as water bodies in step 1 using the features: maximum pixel value (Jul Landsat band 1); mean (Jul Landsat band 5, ASTER band 2); standard deviation (Jul Landsat band 7); and maximum difference.

- - 1. **Classification of specific land cover classes – classification at level 2**

The final level of classification made use of each of the previous classifications. Objects where the super-object (e.g. level 1 object) was classified as water bodies were also classified as water bodies. Objects where the super-object was classified as non-vegetated land were classified as artificial and bare ground. Training data were used to create samples (overlap parameter set to 0.1) for the remaining classes (swamp, rice, crops and grassland, woodland and shrubs). The feature space optimisation tool was used to select the features which resulted in the best separation of classes, and nearest neighbour classification was performed using these features on objects which were not classified as water bodies or artificial and bare ground. The features used were: area; mean difference to neighbours (Dec Landsat band 2 and ASTER band 2); maximum pixel value (Dec Landsat band 4, Jul Landsat bands 4 and 7); mean (Jul Landsat band 2); standard deviation (Dec Landsat bands 4 and 5); mode (Jul Landsat band 2); minimum pixel value (ASTER band 1 and Dec Landsat band 2); ratio (ASTER band 3 and Jul Landsat band 3). Finally, manual editing was performed on some areas where there were unclassified objects or inconsistencies (e.g. the tops of hills) based on visual interpretation of the remotely sensed imagery and the classification of surrounding objects.

- 1. **Accuracy assessment**

See Table S3 for a confusion matrix for the flooding versus non-flooding land classification, Table S4 for a confusion matrix for the vegetated versus non-vegetated land classification and Table S5 for a confusion matrix for the final classification. The results show user and producer accuracies of greater than 0.7 for all classes, with the majority of classes having accuracies of greater than 0.8. This indicates a high level of classification accuracy.

**Table C: Confusion matrix for flooding and non-flooding land classification. Overall accuracy = 0.92.**

|  | Not flooding | Flooding |
| --- | --- | --- |
| Not flooding | 922 | 51 |
| Flooding | 5 | 144 |
| Unclassified | 41 | 0 |
|  |  |  |
| Producer accuracy | 0.95 | 0.74 |
| User accuracy | 0.95 | 0.97 |

**Table D: Confusion matrix for vegetated and non-vegetated land classification. Overall accuracy = 0.99**

|  | Vegetated | Not vegetated |
| --- | --- | --- |
| Vegetated | 1100 | 11 |
| Not vegetated | 6 | 46 |
| Unclassified | 0 | 0 |
|  |  |  |
| Producer accuracy | 0.99 | 0.81 |
| User accuracy | 0.99 | 0.88 |

**Table E: Confusion matrix for final classification. Overall accuracy = 0.88**

|  | Artificial and bare ground | Woodland and shrubs | Crops and grassland | Rice | Swamp |
| --- | --- | --- | --- | --- | --- |
| Artificial and bare ground | 55 | 1 | 7 | 0 | 0 |
| Woodland and shrubs | 0 | 203 | 63 | 0 | 3 |
| Crops and grassland | 11 | 11 | 610 | 0 | 8 |
| Rice | 0 | 0 | 13 | 91 | 0 |
| Swamp | 0 | 16 | 8 | 0 | 58 |
| Unclassified | 0 | 0 | 0 | 0 | 0 |
|  |  |  |  |  |  |
| Producer accuracy | 0.83 | 0.88 | 0.87 | 1.0 | 0.84 |
| User accuracy | 0.87 | 0.75 | 0.95 | 0.88 | 0.71 |

1. **Covariate data**

Several variables were selected from the household level, individual human and individual pig questionnaire data which were thought to be relevant to the risk of *Taenia* spp. infections, based on prior understanding of the disease transmission cycle and previously published research. These variables are listed in Table 1 in the manuscript. A land cover map was derived from remote sensed satellite imagery, as described above. The land cover classification was carried out in a hierarchical manner, resulting in two overarching classes (a) vegetated land (versus built up and bare ground) and (b) flooding land; and four lower level classes (c) agricultural land and grassland, (d) swamp, (e) trees and shrubs, and (f) water bodies. An additional, aggregate class, (g) flooding agricultural land and grassland, was created via an overlay of the flooding land with the agricultural land and grassland classes. Buffers of 1 km were created around each study homestead and the percentage of each land cover type within the buffer was calculated using ArcMap 10 (ESRI, Redlands), to represent the landscape surrounding each homestead. This buffer size was selected to ensure the full landscape in which humans and pigs may be exposed to *Taenia* spp. eggs was included. Previous research has indicated that free-ranging pigs (in western Kenya) move an average of over 4 km per day ([1](#_ENREF_1)), and that human short-term daily average trip distances (in neighbouring Uganda) in a rural village setting are approximately 3 km ([2](#_ENREF_2)).

Temperature (minimum, maximum and mean) and precipitation data were obtained from the Worldclim dataset at a spatial resolution of 1 km ([3](#_ENREF_3)): only mean temperature was considered in the analyses as the ranges of values present across the study area for minimum and maximum temperature were small. Interpolated soil properties (sand content and pH [measured in water]) were obtained from the International Soil Reference and Information Centre at a spatial resolution of 1 km ([4](#_ENREF_4)). Population density data were obtained from the World Pop project at a spatial resolution of 100 m ([5](#_ENREF_5)) and elevation data were obtained from the Shuttle Radar Topography Mission (SRTM) at a spatial resolution of 90 m ([6](#_ENREF_6)). Temperature, precipitation, soil information, elevation and population density data were linked to the survey households by geographical location.

1. Thomas LF, de Glanville WA, Cook EA, Fevre EM. The spatial ecology of free-ranging domestic pigs (Sus scrofa) in western Kenya. Bmc Veterinary Research. 2013;9.

2. Bryceson DF, Mbara TC, Maunder D. Livelihoods, daily mobility and poverty in sub-saharan Africa. Transport Reviews. 2003;23(2):177-96.

3. Hijmans RJ, Cameron SE, Parra JL, Jones PG, Jarvis A. Very high resolution interpolated climate surfaces for global land areas. International Journal of Climatology. 2005;25:1965-78.

4. Hengl T, Heuvelink GBM, Kempen B. Soil property maps of Africa at 1 km. available from [www.isric.org:](http://www.isric.org:) ISRIC: World Soil Information; 2013.

5. Worldpop alpha version 2010 estimates of numbers of people per grid square. <http://www.worldpop.org.uk/data/summary/?contselect=Africa&countselect=Kenya&typeselect=Population2010>.

6. Jarvis A, Reuter HI, Nelson A, Guevara E. Hole-filled seamless SRTM data V4. available from <http://srtm.csi.cgiar.org:> International Centre for Tropical Agriculture (CIAT); 2008.
