# Supplementary material for: The Influence of Socio-economic, Behavioural and Environmental Factors on Taenia spp. Transmission in Western Kenya: Evidence from a Cross-Sectional Survey in Humans and Pigs
Source: PLoS Negl Trop Dis. 2015 Dec 7;9(12):e0004223. doi: 10.1371/journal.pntd.0004223 (PMC4671581; doi:10.1371/journal.pntd.0004223)
Supplement: S3 File — (DOCX) [file pntd.0004223.s004.docx]

**Supporting information 3: Exploratory analysis results.**

**Table A:** Model comparison for different functional forms of land cover and precipitation variables for presence of *Taenia* spp. antigen in humans. *Statistically significant improvement in model fit using a non-linear form.

| **Covariate** | **Form** | **Regression p-value** | **Regression AIC** | **Anova p-value (vs linear form)** |
| --- | --- | --- | --- | --- |
| **% agricultural land and grassland** | **Linear** | 0.07 | 879.9 |  |
|  | **Quadratic** | 0.92;  0.82 | 881.8 | 0.99 |
|  | **Square root** | 0.07 | 879.9 | 0.99 |
|  | **Log** | 0.08 | 879.9 | 0.99 |
| **% flooding land** | **Linear** | 0.30 | 882.3 |  |
|  | **Quadratic** | 0.09 | 882.1 | 0.55 |
|  | **Square root** | 0.36 | 882.5 | 0.99 |
|  | **Log** | 0.55 | 882.8 | 0.99 |
| **% flooding agricultural land and grassland** | **Linear** | 0.19 | 881.7 |  |
|  | **Quadratic** | 0.003;  0.01 | 875.2 | 0.04* |
|  | **Square root** | 0.17 | 881.5 | 0.98 |
|  | **Log** | 0.17 | 881.4 | 0.97 |
| **% swamp** | **Linear** | 0.27 | 882.0 |  |
|  | **Quadratic** | 0.36;  0.70 | 883.9 | 0.99 |
|  | **Square root** | 0.22 | 881.8 | 0.98 |
|  | **Log** | 0.20 | 881.7 | 0.96 |
| **% woodland and shrubs** | **Linear** | 0.07 | 880 |  |
|  | **Quadratic** | 0.90;  0.57 | 881.7 | 0.95 |
|  | **Square root** | 0.11 | 880.7 | 0.99 |
|  | **Log** | 0.19 | 881.6 | 0.99 |
| **% vegetated land** | **Linear** | 0.27 | 882.1 |  |
|  | **Quadratic** | 0.53;  0.46 | 883.6 | 0.90 |
|  | **Square root** | 0.29 | 882.2 | 0.99 |
|  | **Log** | 0.32 | 882.4 | 0.99 |
| **% water bodies** | **Linear** | 0.68 | 883.1 |  |
|  | **Quadratic** | 0.92;  0.97 | 885.1 | 0.99 |
|  | **Square root** | 0.59 | 883.0 | 0.99 |
|  | **Log** | 0.59 | 883.0 | 0.99 |
| **Precipitation** | **Linear** | 0.01 | 877 |  |
|  | **Quadratic** | 0.99;  0.87 | 879.0 | 0.99 |
|  | **Square root** | 0.01 | 877.1 | 0.99 |
|  | **Log** | 0.01 | 877.1 | 0.99 |

**Table B:** Model comparison for different functional forms of land cover and precipitation variables for presence of *Taenia* spp. antigen in pigs. *Statistically significant improvement in model fit using a non-linear form.

| **Covariate** | **Form** | **Regression p-value** | **Regression AIC** | **Anova p-value (vs linear form)** |
| --- | --- | --- | --- | --- |
| **% agricultural land and grassland** | **Linear** | 0.10 | 86.49 |  |
|  | **Quadratic** | 0.57;  0.28 | 87.46 | 0.60 |
|  | **Square root** | 0.12 | 86.75 | 0.99 |
|  | **Log** | 0.15 | 86.98 | 0.99 |
| **% flooding land** | **Linear** | 0.03 | 84.59 |  |
|  | **Quadratic** | 0.53;  0.22 | 84.94 | 0.44 |
|  | **Square root** | 0.07 | 86.183 | 0.99 |
|  | **Log** | 0.16 | 87.37 | 0.99 |
| **% flooding agricultural land and grassland** | **Linear** | 0.009 | 81.76 |  |
|  | **Quadratic** | 0.66;  0.43 | 81.79 | 0.37 |
|  | **Square root** | 0.03 | 84.632 | 0.99 |
|  | **Log** | 0.10 | 86.545 | 0.99 |
| **% swamp** | **Linear** | 0.61 | 89.1 |  |
|  | **Quadratic** | 0.70;  0.63 | 90.69 | 0.81 |
|  | **Square root** | 0.61 | 89.13 | 0.99 |
|  | **Log** | 0.74 | 89.29 | 0.99 |
| **% woodland and shrubs** | **Linear** | 0.41 | 88.7 |  |
|  | **Quadratic** | 0.03;  0.03 | 86.43 | 0.12 |
|  | **Square root** | 0.11 | 86.88 | 0.40 |
|  | **Log** | 0.06 | 85.59 | 0.21 |
| **% vegetated land** | **Linear** | 0.25 | 87.65 |  |
|  | **Quadratic** | 0.68;  0.71 | 89.49 | 0.92 |
|  | **Square root** | 0.25 | 87.63 | 0.99 |
|  | **Log** | 0.25 | 87.60 | 0.98 |
| **% water bodies** | **Linear** | 0.69 | 89.24 |  |
|  | **Quadratic** | 0.28;  0.54 | 88.26 | 0.22 |
|  | **Square root** | 0.30 | 88.42 | 0.66 |
|  | **Log** | 0.35 | 88.59 | 0.72 |
| **Precipitation** | **Linear** | 0.19 | 87.695 |  |
|  | **Quadratic** | 0.15;  0.17 | 87.732 | 0.37 |
|  | **Square root** | 0.17 | 87.58 | 0.94 |
|  | **Log** | 0.16 | 87.45 | 0.88 |

**Table C:** Univariate multilevel model results for presence of *Taenia* spp. antigen in humans – level 1 (individual level covariates). *Instability in model estimates, thus, these are not displayed.

| **Level 1 covariates** | **Estimate** | **SE** | **p-value** |
| --- | --- | --- | --- |
| **Age group (5-14 is ref)** |  |  |  |
| 15 – 24 | -0.07 | 0.33 | 0.82 |
| 25 – 39 | -0.06 | 0.32 | 0.85 |
| 40 – 59 | 0.04 | 0.35 | 0.91 |
| 60 + | 0.77 | 0.40 | **0.06** |
| **Gender (female is ref)** |  |  |  |
| Male | -0.55 | 0.23 | **0.02** |
| **Tribe (Luhya is ref)** |  |  |  |
| Luo | 0.46 | 0.39 | 0.24 |
| Samia | 0.48 | 0.51 | 0.35 |
| Teso | 0.84 | 0.46 | **0.07** |
| Other | -1.02 | 1.46 | 0.48 |
| **Religion (Muslim is ref)** |  |  |  |
| Christian | * |  |  |
| None | * |  |  |
| Other | * |  |  |
| **Education (none is ref)** |  |  |  |
| Primary | -0.54 | 0.28 | **0.05** |
| Secondary | -0.50 | 0.44 | 0.26 |
| Above | -0.30 | 0.71 | 0.67 |
| **Eat beef (no is ref)** |  |  |  |
| Yes | 0.13 | 0.33 | 0.68 |
| **Frequency of beef (weekly is ref)** |  |  |  |
| Monthly or less often | 0.17 | 0.28 | 0.53 |
| Never | -0.04 | 0.36 | 0.91 |
| **Eat pork (no is ref)** |  |  |  |
| Yes | -0.03 | 0.25 | 0.92 |
| **Frequency of pork (weekly is ref)** |  |  |  |
| Monthly or less often | -0.19 | 0.34 | 0.56 |
| Never | -0.13 | 0.37 | 0.72 |
| **Frequency using latrine (always is ref)** |  |  |  |
| Frequently | -0.52 | 0.34 | 0.12 |
| Sometimes | -0.09 | 0.37 | 0.82 |
| Never | 0.22 | 0.52 | 0.67 |

**Table D:** Univariate multilevel model results for presence of *Taenia* spp. antigen in humans – level 2 (household level covariates). Water source results provided relate to the wet season: results for dry season water sources were comparable to those presented.

| **Level 2 covariates** | **Estimate** | **SE** | **p-value** |
| --- | --- | --- | --- |
| **Latrine in compound (no is ref)** |  |  |  |
| Yes | -0.18 | 0.43 | 0.67 |
| **Latrine type (completely closed is ref)** |  |  |  |
| Partially closed | -0.31 | 0.46 | 0.5 |
| Open pit | -0.53 | 0.95 | 0.58 |
| None | -0.05 | 0.53 | 0.92 |
| **Evidence of latrine use (no is ref)** |  |  |  |
| Yes | -0.42 | 1.14 | 0.71 |
| No latrine | -0.23 | 1.18 | 0.85 |
| **Recent village flooding (no is ref)** |  |  |  |
| Yes | 0.79 | 0.44 | **0.07** |
| **Pig keeping (no is ref)** |  |  |  |
| Yes | -0.69 | 0.50 | 0.16 |
| **Well water (no is ref)** |  |  |  |
| Yes | 1.16 | 0.47 | **0.01** |
| **Use roof water (no is ref)** |  |  |  |
| Yes | 0.12 | 0.37 | 0.74 |
| **Use river water (no is ref)** |  |  |  |
| Yes | -0.22 | 0.48 | 0.65 |
| **Use piped water (no is ref)** |  |  |  |
| Yes | -1.32 | 0.85 | 0.12 |
| **Use dam/pond water (no is ref)** |  |  |  |
| Yes | -1.09 | 1.11 | 0.33 |
| **Use borehole water (no is ref)** |  |  |  |
| Yes | -0.05 | 0.37 | 0.89 |
| **Use spring water (no is ref)** |  |  |  |
| Yes | -0.83 | 0.37 | **0.02** |
| **% agricultural land and grassland** | 0.03 | 0.02 | **0.09** |
| **% flooding land** | 0.007 | 0.008 | 0.40 |
| **% flooding agricultural land and grassland** |  |  |  |
| **% flooding agricultural land and grassland**  **% flooding agricultural land and grassland^2^** | 11.22  -22.98 | 3.84  9.36 | **0.003**  **0.01** |
| **% swamp** | -0.03 | 0.03 | 0.29 |
| **% swamp**  **% swamp^2^** |  |  |  |
| **% woodland and shrubs** | -0.03 | 0.02 | 0.12 |
| **% vegetated land (vs bare or built up)** | -0.02 | 0.02 | 0.33 |
| **% water bodies** | -0.02 | 0.05 | 0.73 |
| **Soil sand content** | -0.10 | 0.02 | 0.67 |
| **Water pH** | 0.17 | 0.28 | 0.55 |
| **Mean temperature** | 0.05 | 0.06 | 0.38 |
| **Precipitation** | -0.002 | 0.0009 | **0.05** |
| **Elevation** | -0.002 | 0.003 | 0.60 |
| **Population density** | -0.06 | 0.05 | 0.24 |

**Table E:** Univariate single-level model results for presence of *Taenia* spp. antigen in pigs.

| **Covariates** | **Estimate** | **SE** | **p-value** |
| --- | --- | --- | --- |
| **Age group (<4 months is ref)** |  |  |  |
| 4 – 12 months | 15.9 | 1398.7 | 0.99 |
| >12 months | 17.10 | 1398.7 | 0.99 |
| **Gender (female is ref)** |  |  |  |
| Male | -0.33 | 0.56 | 0.56 |
| **Origin (born in homestead is ref)** |  |  |  |
| External | -0.26 | 0.60 | 0.67 |
| **Gender and breeding status (male is ref)** |  |  |  |
| Non-breeding sow | -0.11 | 0.62 | 0.86 |
| Breeding sow | 2.05 | 0.88 | **0.02** |
| **Sheep kept (no is ref)** |  |  |  |
| Yes | 1.12 | 0.57 | **0.05** |
| **Goats kept (no is ref)** |  |  |  |
| Yes | 0.12 | 0.55 | 0.83 |
| **Latrine in compound (no is ref)** |  |  |  |
| Yes | 0.55 | 1.10 | 0.61 |
| **Latrine type (completely closed is ref)** |  |  |  |
| Partially closed | 1.39 | 1.08 | 0.20 |
| Open pit | 2.77 | 1.75 | 0.11 |
| None | 0.69 | 1.48 | 0.64 |
| **Latrine scavenging (no latrine is ref)** |  |  |  |
| No | 0.57 | 1.10 | 0.61 |
| Yes | 0.47 | 1.31 | 0.72 |
| **Recent village flooding (no is ref)** |  |  |  |
| Yes | 0.21 | 0.84 | 0.80 |
| **Sell piglets (no is ref)** |  |  |  |
| Yes | 1.12 | 0.80 | 0.16 |
| **Raise pigs for meat (no is ref)** |  |  |  |
| Yes | -0.53 | 0.61 | 0.38 |
| **% agricultural land and grassland** | 0.04 | 0.03 | **0.10** |
| **% flooding land** | 0.02 | 0.009 | **0.03** |
| **% flooding agricultural land and grassland** | 0.03 | 0.01 | **0.009** |
| **% swamp** | -0.02 | 0.05 | 0.61 |
| **% woodland and shrubs** | -0.03 | 0.03 | 0.41 |
| **% vegetated land (vs bare and built up)** | 0.07 | 0.06 | 0.25 |
| **% water bodies** | 0.06 | 0.16 | 0.69 |
| **Soil sand content** | 0.007 | 0.04 | 0.85 |
| **Water pH** | 2.19 | 0.99 | **0.03** |
| **Mean temperature** | 0.10 | 0.11 | 0.37 |
| **Precipitation** | -0.001 | 0.001 | 0.19 |
| **Elevation** | -0.008 | 0.006 | 0.17 |
| **Population density** | -0.50 | 0.44 | 0.26 |
